# Putting life on ice: bacteria that bind to frozen water

**DOI:** 10.1098/rsif.2016.0210

**Published:** 2016-08

**Authors:** Maya Bar Dolev, Reut Bernheim, Shuaiqi Guo, Peter L. Davies, Ido Braslavsky

**Affiliations:** 1 Institute of Biochemistry, Food Science and Nutrition, The Robert H. Smith Faculty of Agriculture, Food and Environment, The Hebrew University of Jerusalem, Rehovot 7610001, Israel; 2 Department of Biomedical and Molecular Sciences, Queen's University, Kingston, Ontario, Canada K7L 3N6

**Keywords:** ice-binding proteins, antifreeze proteins, biofilm, RTX adhesin, microfluidic cold finger, cold adaptation

## Abstract

Ice-binding proteins (IBPs) are typically small, soluble proteins produced by cold-adapted organisms to help them avoid ice damage by either resisting or tolerating freezing. By contrast, the IBP of the Antarctic bacterium *Marinomonas primoryensis* is an extremely long, 1.5 MDa protein consisting of five different regions. The fourth region, a 34 kDa domain, is the only part that confers ice binding. Bioinformatic studies suggest that this IBP serves as an adhesin that attaches the bacteria to ice to keep it near the top of the water column, where oxygen and nutrients are available. Using temperature-controlled cells and a microfluidic apparatus, we show that *M. primoryensis* adheres to ice and is only released when melting occurs. Binding is dependent on the mobility of the bacterium and the functionality of the IBP domain. A polyclonal antibody raised against the IBP region blocks bacterial ice adhesion. This concept may be the basis for blocking biofilm formation in other bacteria, including pathogens. Currently, this IBP is the only known example of an adhesin that has evolved to bind ice.

## 1. Introduction

Many organisms living with the threat of freezing in cold ecosystems have adapted to these harsh conditions by producing ice-binding proteins (IBPs). IBPs adsorb to ice surfaces to serve different biological roles [1,2]. Small (3–30 kDa) IBPs known as antifreeze proteins (AFPs) help organisms avoid freezing by inhibiting the growth of seed ice crystals in their body fluids. Marine fish at high latitudes can encounter sea ice and yet do not freeze because AFPs in their blood lower their freezing temperature by approximately 1°, enough to allow supercooling in the presence of ice. This ice growth inhibition results in depression of the freezing point [3] and is accompanied by a slight elevation of the melting point [4–6]. The consequent difference between the freezing and the melting points is termed thermal hysteresis (TH) and is used to quantify the activity of AFPs and other IBPs *in vitro*.

In comparison with fish, some cold-adapted insects produce ‘hyperactive’ AFPs that can inhibit ice growth by more than 5° due in part to differences in ice plane affinity [7]. IBPs in freeze-tolerant plants are secreted to the extracellular space and act to inhibit ice recrystallization (IR) [8], a phenomenon in which water moves from a large number of small ice crystals to form a small number of large crystals. IR increases ice grain size and thereby causes cell separation and destructive mechanical stress. It has been speculated that IR inhibition is also the primary activity of various microorganism IBPs from bacteria [9,10], sea ice diatoms [11] and Antarctic algae [12,13]. A role suggested for the secreted IBPs is to depress the migration of grain boundaries in snow, sea ice [12] or even ancient glacial ice [14]. In between these boundaries liquid is entrapped, forming a network of veins and sheets rich in nutrients that serves as a habitat for microbial communities [15,16].

IBPs differ not only in their natural function but also in their sequences and structures [1]. They are usually small, single-domain, soluble proteins. However, the IBP isolated from the Gram-negative, aerobic, Antarctic bacterium *Marinomonas primoryensis* (*Mp*IBP) is an exceptionally large protein that consists of five distinct structural regions with a total mass of approximately 1.5 MDa [17,18]. Only the fourth region of this protein (*Mp*IBP_RIV), which is 2% of the whole protein weight, has ice-binding activity [18,19]. *Mp*IBP_RIV is a single, calcium-dependent 34 kDa domain composed of 13 repeats of approximately 19 amino acids that folds into a repetitive β-solenoid structure, as shown by X-ray crystallography [20]. A flat, repetitive two-dimensional array of Thr and Asn residues makes up its ice-binding face. The TH activity level of 0.5 mg ml^−1^ *Mp*IBP_RIV is over 2° [17,18], and it can bind both prismatic and basal planes of ice [21]. The structure, TH levels and basal plane affinity are characteristic of hyperactive AFPs. However, these properties were obtained for recombinant *Mp*IBP_RIV *in vitro,* without the rest of the protein [18]. Most of the size of *Mp*IBP is due to region II (*Mp*IBP_RII), composed of approximately 120 tandem repeats of a 104-amino-acid extender domain. In total, the protein is estimated to be 0.6 µm long if fully folded and extended [22]. Unlike most bacterial IBPs, *Mp*IBP is not secreted into the medium [17].

**Figure 1.**
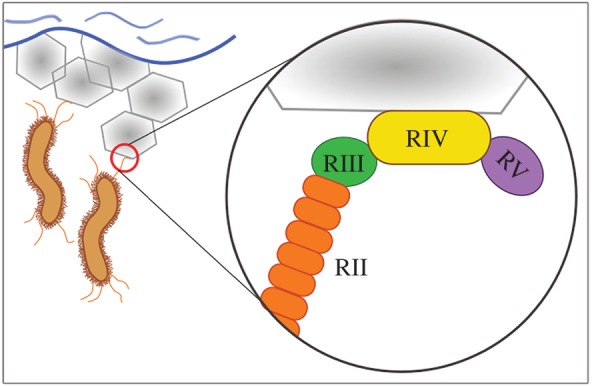
Schematic presentation of *Marinomonas primoryensis* bound to sea ice through *Mp*IBP. Sea ice is represented by the hexagons. An enlargement of the binding location at the C-terminal end of *Mp*IBP shows *Mp*IBP_RIV (yellow), the ice-binding domain, flanked by RIII (green) and RV (purple). Only a few units of RII (out of approximately 120 repeats) are shown (orange). (Figure prepared using Inkscape (open source).)

The extreme size of *Mp*IBP and the fact that 98% of its mass is not related to antifreeze activity led to the notion that this protein serves a distinct function from other IBPs. Bioinformatic analyses suggest that *Mp*IBP is an adhesin and is a type of biofilm-associated protein [19]. Region V at the C-terminal end of the protein contains several repeats-in-toxin units that might be responsible for secretion of *Mp*IBP out of the cell but leaving it bound to the cell surface (figure 1) [19].

In this paper, we present direct evidence for adhesion of *M. primoryensis* bacteria to ice. We demonstrate that the bacteria can adhere to ice as microcolonies and remain in place as long as the ice does not melt. We confirm that *Mp*IBP is located at the bacterial surface and show that antibody raised against *Mp*IBP_RIV blocks binding of the bacteria to ice. We also show that other psychrophilic bacteria, which do not obviously possess an IBP on their surface, do not adhere to ice.

## 2. Material and methods

### 2.1. Material and proteins

Dry media and agar were purchased from BD Difco. Other materials were purchased from Sigma-Aldrich, unless specified otherwise. Polyclonal antibodies were raised in rabbits immunized with recombinant *Mp*IBP_RIV or with a recombinant segment of the *Mp*IBP region II comprising four tandem Ig-like repeats (4xR_II) [19]. The 4xR_II fragment was prepared as previously described [23]. As a control, we used the sera of the same rabbits before they were immunized. Secondary antibodies and sera were purchased from Jackson ImmunoResearch, PA, USA. Recombinant *Mp*IBP_RIV was prepared as described previously [18].

### 2.2. Bacterial strains and growth conditions

*Marinomonas primoryensis* were cultured from frozen glycerol stocks of Antarctic isolates [24], and were grown by incubating 2–3 ml cultures in 0.5× marine broth at 4°C without shaking for 4–5 days. After this period, the culture reached an optical density (OD_600 nm_) of 0.3–0.5. To check the effect of EGTA on *M. primoryensis*, 3 µl of 250 mM EGTA solution (pH 8) was added to 50 µl aliquots of bacterial culture to make a final concentration of 15 mM EGTA. The high concentration was necessary to chelate all calcium ions in the medium. Alternatively, the bacterial culture was centrifuged for 5 min at 2000*g* and the pellet was suspended in 20 mM Tris-HCl (pH 8), 500 mM NaCl and 5 mM EGTA. This bacterial suspension was incubated for 0.5–2 h at 4°C before observation. *Pseudomonas borealis, P. syringae* and the *P. syringae cit7del* mutant were kindly provided by Virginia Walker, Queen's University, Kingston, Ontario, Canada. These strains and *P. fluorescens* (a gift from Dr Roni Shapira, The Hebrew University of Jerusalem, Rehovot, Israel) were grown in 3% or 10% tryptic soy broth (Himemedia, India) for 2–4 days at 4°C without shaking. Other bacterial strains used as controls were gifts from several laboratories. A detailed list of those strains with recommended growth conditions from the donors are given in the electronic supporting material.

### 2.3. Electron microscopy

A culture of *M. primoryensis* was pelleted for 5 min at 4500*g* and washed twice with 0.1 M sodium cacodylate (pH 7.4) supplemented with 5 mM CaCl_2_ (CaCo buffer). The bacteria were fixed in 3% paraformaldehyde (diluted from 16% solution; Electron Microscopy Sciences (EMS), PA, USA) and 2% glutaraldehyde (diluted from 25% solution; EMS) in CaCo buffer for 24 h at 4°C and then washed three times in 3 ml CaCo buffer. The washed bacteria were resuspended in 100 µl CaCo buffer and 1 : 100 diluted bacteria in CaCo buffer were immobilized on polylysine-coated Si wafers (50 µl for each 5 × 5 mm wafer) for 24 h at 4°C. The coated chips were then fixed in 1% osmium tetraoxide solution (diluted from 4% solution; EMS) in CaCo buffer for 1 h at room temperature followed by three washes in 5 ml CaCo buffer. The samples were then dehydrated by two 5 min incubations in increasing amounts of ethanol (25%, 50%, 75% and 96%) followed by two 10 min washes in 100% ethanol. The dehydrated samples were dried by a critical point drying system (K850; Quorum Technologies, UK) and visualized by ultra-high-resolution scanning electron microscopy (ultra-SEM; Ultra55; Zeiss).

### 2.4. Adhesion to ice in suspension

We used a custom-made nanolitre osmometer system to observe the behaviour of bacteria in a solution containing an ice crystal. This system allows delicate manipulation of the temperature of an aqueous droplet, approximately 200 µm in diameter with a precision of a few millikelvin. A detailed description of the system has been given elsewhere [25,26]. In a typical experiment, bacterial cultures (30 µl) were supplemented with 10 µl of serum containing anti-*Mp*IBP_RIV antibody, anti-*Mp*IBP_RII antibody or pre-immunized serum, and incubated at 4°C overnight. Samples were then diluted fivefold in 0.5× marine broth, and ice-nucleating agent (Snomax; Johnson Controls, Milwaukee WI, USA) was added (2–5 µg ml^−1^). In the experiments including AFPs, the samples were also supplemented with 0.1 µM *Tenebrio molitor* AFP (*Tm*AFP) to help stabilize the ice crystal. The samples were injected into the sample holder and handled as in the ice morphology experiments previously described [27]. Briefly, samples were cooled until frozen and then slowly warmed back such that a single ice crystal was left in the solution. This crystal was warmed and melted until it reached the desired size. For experiments with AFPs, when the ice crystal had a diameter of 50–100 µm, the temperature was lowered to a few millikelvin below the melting point to allow the crystal to stabilize. Crystals were observed over time without changing the temperature. When AFP was not present, the crystals were melted to 15–20 µm and the temperature was slightly lowered during the experiment to ensure that no melting occurred. These experiments were conducted at slow ice growth rates.

### 2.5. Analysis of bound bacteria

To count the number of bacteria bound to ice we used low bacterial concentrations, with an OD_600 nm_ of approximately 0.02, corresponding to 1–2 × 10^7^ colony forming units per millilitre (determined by colony counting). Using this concentration, we obtained images with 250–400 bacteria per frame. Higher concentrations resulted in too many bacteria, which were difficult to distinguish and count. Ice crystals were typically followed from 15–20 µm to a diameter of 60 µm, for a period of approximately 10 min. If local melting was observed during this period, the experiment was discarded. This is because local melting resulted in release of bacteria from the surface. On the other hand, if the crystal was growing too fast it exceeded our field of view too quickly and there was not sufficient time for the bacteria to bind. To meet these conditions, we kept the ice growth rates in the range of 0.01–0.04 µm s^−1^ (diameter) by subtle temperature changes with millikelvin resolution. The experiment was stopped when the crystal projected area reached approximately 3400 µm^2^ (radius of approx. 33 µm), corresponding to 47% of the frame so that in all images we were able to see more than 50% of solution area (the total field of view calculated from the image size is 7200 µm^2^). In this way we avoided bias due to too little solution area in the frame. The crystal volume at the end was at least 100-fold smaller than the whole droplet (less than 0.1 nl in droplets of approximately 10–20 nl). Snapshots were taken every minute, and bacteria were manually counted in the solution and on the crystal surface with the aid of electronic registration using ImageJ software [28]. In these experiments, either anti-*Mp*IBP_RIV serum or pre-immune rabbit serum was used to make sure that there was no artefact of the serum influencing the results.

### 2.6. Adhesion to ice in microfluidic systems

**Figure 2.**
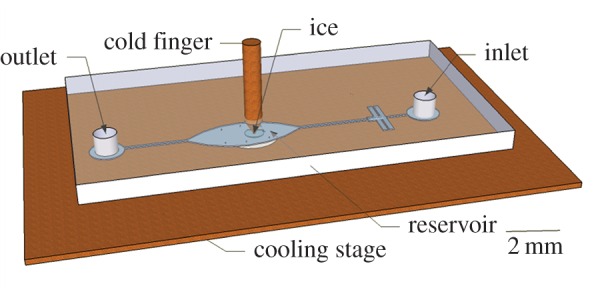
An illustration of the MCF. The microfluidic chip is placed on top of a cooling stage. A copper cold finger embedded in the middle of the channel functions as an independent cooling unit. An ice crystal is drawn as a circle in the channel below the tip of the cold finger. (Figure prepared using Google Sketchup.)

We used a custom-designed temperature-controlled cell that allows local growth of ice by formation of a temperature gradient, as shown in figure 2. We named this apparatus the microfluidic cold finger (MCF) device. A detailed description of the system is given elsewhere [29]. The control over the temperature in the MCF is both from the stage below the microfluidic chip and from the centre of a chamber located in the middle of the microfluidic channel. A copper wire embedded in the middle of the fluid-filled chamber serves as the second temperature-controlled locus around which ice is grown. Two controllers (models 3150 and 3040; Newport Corporation, CA, USA) are used to separately control the temperature from the stage and the cold finger, both with millikelvin resolution. The channel was filled with double-distilled water and cooled until freezing (around −20°C) by cooling both the cold finger and the stage. After freezing, the temperature of the stage was increased to approximately +1°C while the copper wire was kept a few degrees below zero. This process created a temperature gradient across the microfluidic channel, which allowed melting of most of the ice except for that immediately around the cold finger. At this point, *M. primoryensis* were injected into the microfluidic device. Once the device was filled with bacteria the flow was stopped and the bacteria next to the ice front were observed using a Nikon Eclipse Ti inverted microscope equipped with an Intenslight C-HGFIE fibre illuminator (Nikon, Tokyo, Japan). Experiments were recorded using a Neo sCMOS camera (Andor Technology, Belfast, UK).

### 2.7. Immunofluorescence labelling of M*p*IBP_RIV

Cultures (5–10 ml) of *M. primoryensis* were centrifuged for 5 min at 5000*g*. The pellet was resuspended in 1.5 ml of buffer containing 10 mM Tris-HCl (pH 8.0), 140 mM NaCl and 1 mM CaCl_2_ (buffer Ca+) and washed twice by repeating the centrifugation and resuspension process. The pellet was then resuspended in 4% paraformaldehyde (EMS) in buffer Ca+ for 10 min for fixation, and washed twice as above. After the second wash, the pellet was resuspended in 150 µl of buffer Ca+. The fixed bacteria were immobilized by placing 15 µl aliquots on 10 × 10 mm glass coverslips coated with poly-l-lysine. After three washes with the same buffer without calcium (buffer Ca−), the samples were blocked with blocking buffer, 10% goat serum (Jackson ImmunoResearch, PA, USA) in buffer Ca−, overnight at 4°C. The following day the blocking solution was removed and a solution of 1 : 500 anti-*Mp*IBP_RIV antibody in blocking buffer+0.1% Triton 100 was placed on the coverslips for 1 h at room temperature. As a control, we used serum from the same rabbit before immunization. The coverslips were washed three times in Ca− buffer and 1 : 1000 Alexa Fluor^®^488-AffiniPure Goat Anti-Rabbit IgG (H + L) (Jackson ImmunoResearch, PA, USA) was added. After incubation for 1 h, the coverslips were washed in double-distilled water and mounted on glass slides using immune-mount reagent (Thermo Fisher Scientific, MI, USA). Images were taken using an Olympus dp71 camera connected to an Olympus ix51 fluorescence microscope.

## 3. Results

### 3.1. Direct evidence of *Marinomonas primoryensis* adhesion to ice crystals

**Figure 3.**
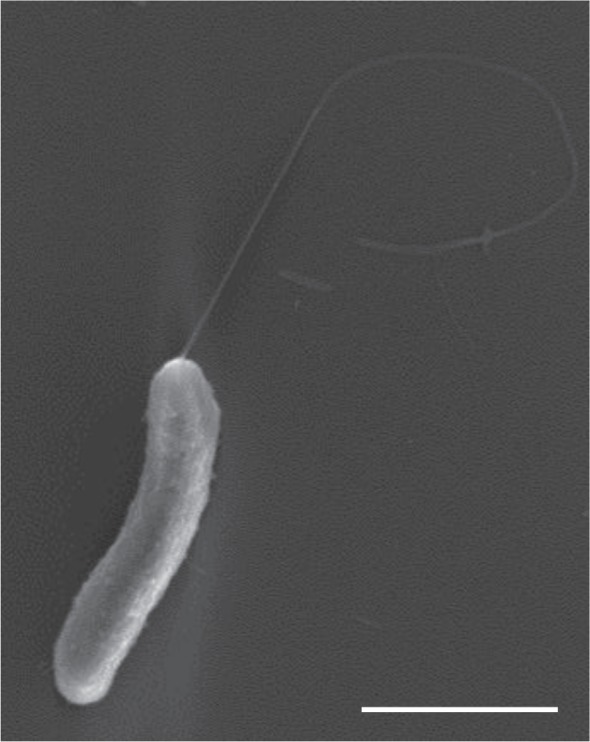
*Marinomonas primoryensis* viewed by ultra-SEM. Scale bar, 1 µm.

*Marinomonas primoryensis* observed by electron microscopy are typically 2–5 µm long with a single polar flagellum (figure 3). Although the flagellum (diameter approximately 30 nm) is clear, there is no sign of other distinct appendages emanating from the surface of the outer membrane. The bacteria are extremely motile at temperatures close to zero with a typical velocity of 20 µm s^−1^ at 0°C, as observed in the nanolitre osmometer (electronic supplementary material, movie S1) and the MCF device (electronic supplementary material, movie S2). Although a low percentage of bacteria survived freezing at approximately −30°C (less than 10%), we found that freezing survival was dramatically improved (more than 90%) by the addition of an ice-nucleating agent (Snomax) that elevated the freezing temperature to around −10°C.

**Figure 4.**
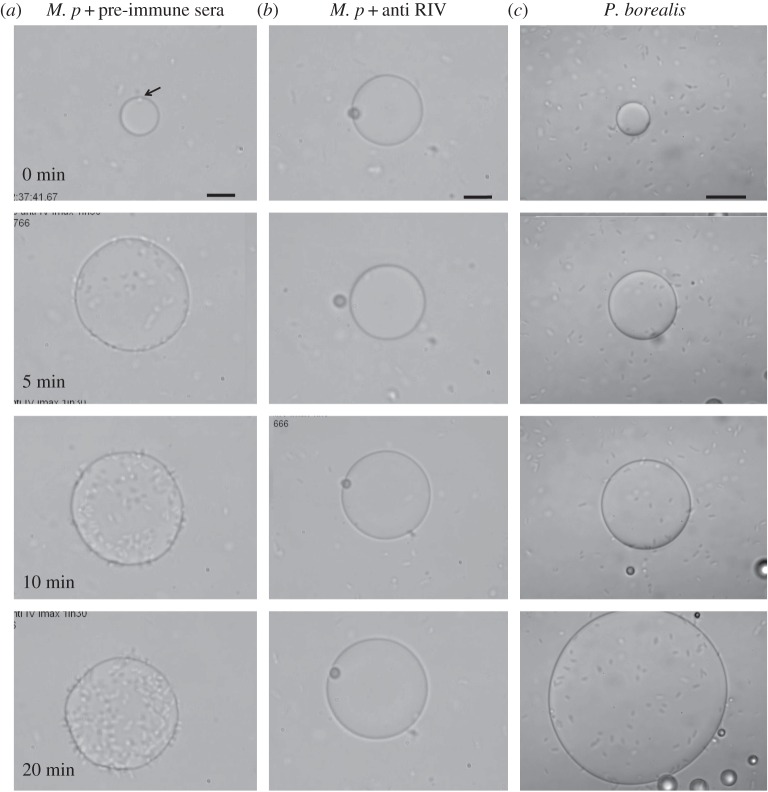
Accumulation of *Marinomonas primoryensis* on ice and blockage of this process by antibody to *Mp*IBP_RIV. (*a*) Time-lapse images of ice grown in medium containing *M. primoryensis* in addition to rabbit pre-immune serum. (*b*) The same sequence of images as in (*a*) but with rabbit anti-*Mp*IBP_RIV serum in place of the pre-immune serum. (*c*) Control series with *Pseudomonas borealis* in place of *M. primoryensis.* Bacteria in the crystal field in (*c*) are not attached to the ice (see the electronic supplementary material, movie S1). Scale bars, 10 µm.

The electronic supplementary material, movie S1, and figure 4*a* present a single ice crystal grown in solution containing a diluted *M. primoryensis* culture*.* The crystal at the beginning is clear and smooth, with only a single bacterium bound to its surface (indicated by the arrow). In the figure, only a few bacteria are visible around the crystal due to the low cell number and the limited focus depth, but their presence is clear in the movie. After 5 min, a few tens of bacteria are already concentrated on the surface of the crystal, and after 20 min much of the crystal is covered by a layer of bacteria. As the whole sample contains approximately 10–20 nl of solution, with just a few hundred bacteria, it seems that most of them have bound to the crystal by this time.

The motility of *M. primoryensis* is crucial for finding and binding ice. Non-motile bacteria that move by Brownian motion in a drop more than 100 µm in diameter are unlikely to encounter an ice crystal that occupies 1% of the fluid volume. A particle 1 µm in diameter moving by Brownian diffusion in water at 0°C will take more than 6 h to travel a distance of 100 µm [30]. Still, it is not clear *a priori* that the binding of *M. primoryensis* to ice is due to its IBP. We cultured other psychrophilic bacteria and motile marine bacteria to test their affinity for ice (described in the electronic supplementary material). Of these bacteria, only the *Pseudomonas* species could swim at close to 0°C. These include *P. borealis* [31,32] and *P. syringae* (INP+) [33], both of which contain ice-nucleating protein (INP) on their outer membranes, *P. syringae cit7del* mutant (INP−), which lacks ice-nucleating activity [34]*,* and *P. fluorescens*, which was suggested to express both INP and AFP [35]. *Pseudomonas borealis* freely swim in the solution but do not bind the ice or sense it in any visible way (figure 4*c*; electronic supplementary material, movie S3). Bacteria that appear to be on the ice surface in figure 4*c* are either swimming above the crystal or are non-motile bacteria entrapped by the growing ice, or floating on top of it. Similar results were obtained with INP− *P. syringae* (cit7del mutant) and with *P. fluorescens* (not shown). *Pseudomonas borealis* and *P. syringae* were reported to have some IR inhibition activity, and ice-shaping activity was noted for *P. borealis* [36]. It is clear from our findings that the putative ice-binding agent of these strains is different from *Mp*IBP in role and activity. We did not observe ice shaping by *P. borealis*, possibly due to the low concentration of bacteria used in our study (figure 4*c*).

The electronic supplementary material, figure S1 (accompanied by electronic supplementary material, movie S4), presents time-lapse snapshots of ice crystals grown in the presence of small amounts (up to 1 µM) of AFP in addition to the bacteria. By adding free *Tm*AFP to the solution, we were able to control ice growth better and inspect stable ice crystals for long time periods. Although there is competition between the free *Tm*AFP molecules and the bacteria, the free protein molecules did not evidently interfere with the binding of the bacteria to ice. The bacteria continued to bind the crystal for over an hour, covering some planes completely. The preferential binding to certain ice faces may be due to the position of the crystal, floating with one plane in contact with the air–water interface, where it is less accessible. Another possibility is that this plane is significantly less preferred for binding by *Mp*IBP, relative to the other covered planes. It might also be that adsorbed *Tm*AFP molecules hinder access of the *M. primoryensis* to it, although at these low protein concentrations (corresponding to TH of less than 0.1°C) the distance between *Tm*AFP molecules should be enough for bacteria to bind [37]. In many other cases when the crystal was not faceted, we observed bacteria covering the ice from all sides, creating a layer of closely contacting bacteria. Replacing *Tm*AFP with spruce budworm antifreeze protein (sbwAFP) or snow flea antifreeze protein (sfAFP) did not change the ability of the bacteria to cover the crystal from all directions. When an ice crystal covered with bacteria was melted, as shown in the electronic supplementary material, movie S4, the bacteria released to the solution began to swim as before. This process was repeated several times with the same sample giving the same outcome.

### 3.2. Adhesion to ice in a microfluidic device

**Figure 5.**
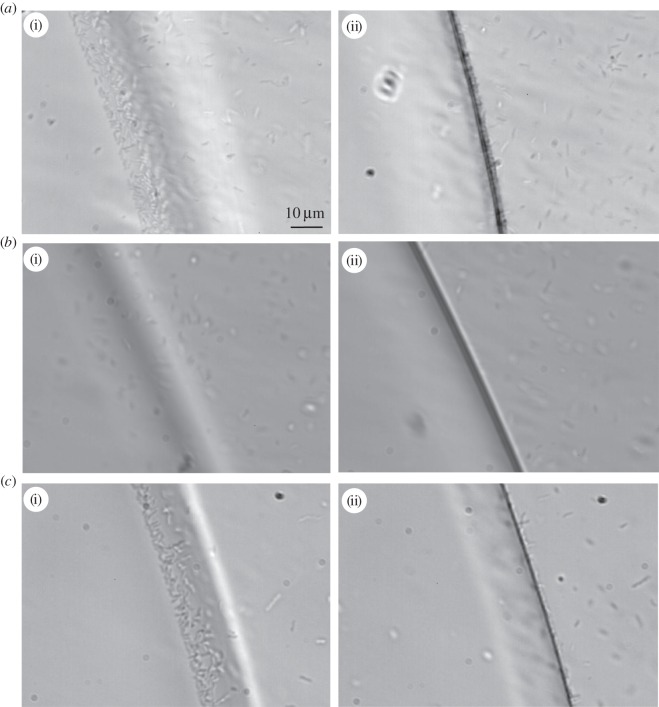
Accumulation of *Marinomonas primoryensis* on ice in microfluidics and the effects of anti-*Mp*IBP_RIV and anti-*Mp*IBP_RII. (*a*) Ice was held at a constant temperature slightly below the melting point in a solution containing fresh bacteria (OD ∼ 0.5) for 3 min before the image was taken. (*b*) Ice was held for 20 min at a constant temperature slightly below the melting point in a solution of bacteria treated overnight with anti-*Mp*IBP_RIV serum (25% v/v). (*c*) Ice was held for 5 min at a constant temperature slightly below the melting point in a solution of bacteria treated overnight with anti-*Mp*IBP_RII serum (25% v/v). The images in (i) and (ii) are focused on the top and bottom layers of the ice, respectively.

To make sure that the ice-binding effect is not influenced by the ice nucleators in the solution, we inspected the ice binding of *M. primoryensis* using microfluidics in the MCF apparatus (described in the Material and methods section), where we could grow an ice crystal in pure water in the middle of the main chamber (figure 2) before injecting bacteria. This allowed us to inspect the behaviour of *M. primoryensis* in the presence of ice without the need for any additive, without changing their growth medium, and without freeze–thaw cycles. Figure 5 and the electronic supplementary material, movie S2, present bacteria that are attaching to a growing ice surface. A monolayer of bacteria covers the ice after a few minutes of incubation. This layer was stable on the ice for at least 1 h and once the crystal was melted the bacteria dispersed and then returned to swimming randomly in solution.

### 3.3. Adhesion to ice by a single point

The electronic supplementary material, movies S1 and S2, demonstrates bacteria freely swimming in solution and binding to ice when they encounter the crystal. Not every time a bacterium encounters the ice does it adhere to it, but, when it binds, the bacterium is rarely detached, implying strong attachment. Many bacteria are bound to ice from one of their poles and they continue to move their bodies after binding. A few bound individuals rotate around their attachment point, indicating that they are bound to the ice by a single connection. This single connection is sufficient to hold the bacterium on the ice surface. Binding through more than one connection would presumably stop the rotation and reduce the tendency for detachment. In most cases the bacteria were not rotating, suggesting multiple connections had been made to the surface.

### 3.4. *Marinomonas primoryensis* has ice-shaping activity

**Figure 6.**
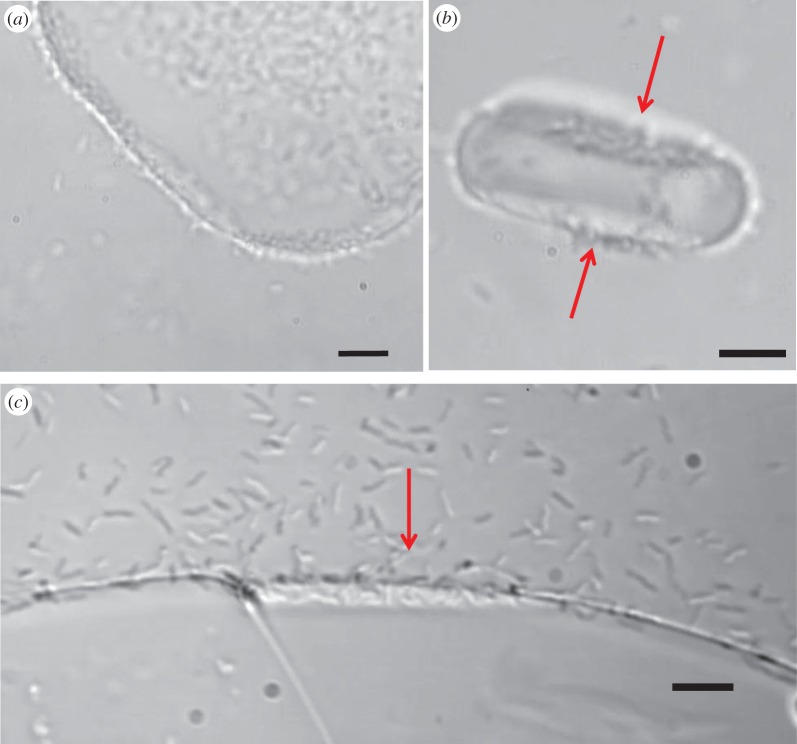
Ice shaping and basal plane affinity by *Marinomonas primoryensis*. (*a*) Extensive accumulation of *M. primoryensis* on the ice surface changes crystal growth morphology from a flat disc to a more hexagonal shape. (*b*) A single ice crystal disc bound by *M. primoryensis* oriented with prism planes edge-on to the camera, showing bacterial accumulation mainly on the basal planes (indicated by red arrows). (*c*) Ice grown in an MCF chamber showing *M. primoryensis* preferentially accumulating on the basal plane (marked by the red arrow). Note: the ice contains more than one grain. The basal plane is identified by its faceting. The rough planes next to it are non-basal planes. Scale bars, 10 µm. (Online version in colour.)

Ice crystals in water or solutions without active IBPs grow as flat discs where the two flat surfaces are the basal planes connected by unfaceted prism surfaces. The crystal shape is altered by active IBPs in various manners depending on their binding plane specificity even at low concentrations. Figure 6*a* shows an ice crystal growing in the presence of *M. primoryensis* at an OD_600 nm_ of 0.5, equivalent to 2–3 × 10^8^ bacteria per ml (calculated by colony count). At these cell concentrations, the amount of *Mp*IBP on the ice crystal is sufficient to prevent growth in particular directions and cause ice shaping into a rounded hexagon. However, the concentrations of bacteria in solution are insufficient to stop the ice from growing.

### 3.5. *Marinomonas primoryensis* binds preferentially to the basal plane of ice

Flat crystals obtained at low concentrations of AFP tend to float with their large, basal planes parallel to the field of view. In figure 6*b*, we caught a crystal rotating during growth just a few seconds after lowering the temperature below the melting point, which allowed us to visualize both the basal and prism planes just after they were formed. It is clear from these images that most of the bacteria are located on the basal planes, while a few are bound to the non-basal surfaces. After longer periods of incubation, the whole crystal is covered with bacteria in all directions. Figure 6*c* demonstrates preferential binding to the basal plane of a large ice crystal grown in the MCF device. We noted that bacteria bound to the basal plane are more static than those on the other planes, where they tend to slide along the ice front.

### 3.6. *Marinomonas primoryensis* are not incorporated into ice but congregate between grain boundaries

**Figure 7.**
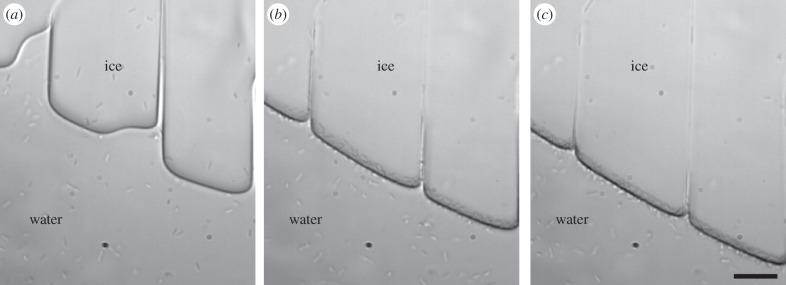
*Marinomonas primoryensis* are not incorporated into growing ice. Images (*a–c*) show advancing ice fronts on three adjacent ice grains growing in the MCF device at a rate of 0.5 µm s^−1^. As the ice fronts advance (from top to bottom), more bacteria accumulate on their surfaces without being engulfed. Scale bars, 10 µm.

**Figure 8.**
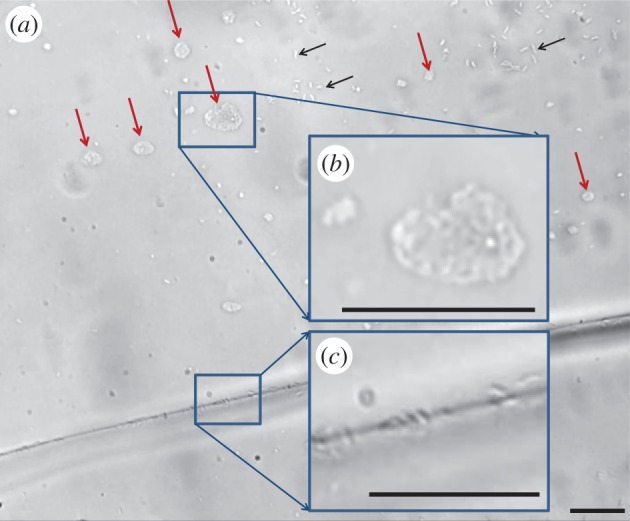
(*a*) Congregation of *Marinomonas primoryensis* on ice. Ice grains in the MCF were grown rapidly in the presence of *M. primoryensis*. Many of the bacteria gathered in microcolonies at the interface between the ice and the microfluidic device (red arrows) or between grain boundaries. Some bacteria remained single on top of the ice (black arrows). (*b*) Magnification of the area in the top rectangle, showing two microcolonies. (*c*) Magnification of the area in the bottom rectangle, showing bacteria between adjacent ice grains. Scale bars, 10 µm.

Figure 7 and the electronic supplementary material, movie S2, show an ice front growing with *M. primoryensis* bound to its surface. As the ice grows, the bacteria move towards the advancing front without being incorporated into the crystal, but many individuals concentrate between grain boundaries. At high supercooling, bacteria can be trapped by fast-growing ice, forming dense patches at the interface with the polydimethylsiloxane walls of the MCF (figure 8 and electronic supplementary material, figure S2). These microcolonies on the ice surface moved as a unit following temperature changes (electronic supplementary material, movie S5), probably due to local flows and temperature gradients [38]. When the ice is melted the bacteria are liberated to the solution and after a few seconds swim as before. The tendency of the bacteria to gather between grain boundaries may result from mechanical forces associated with flow [38]. Gliding in the veins between grains (electronic supplementary material, movie S5) indicates that these bacteria are not bound to the ice. The concentration of nutrients between crystal boundaries can be high due to exclusion of solutes by growing ice and may be beneficial to the bacteria. Similar gliding and accumulation in grain boundaries and patches was observed in solutions of *P. syringae* and *P. borealis*, suggesting that these phenomena are not related to the adhesion protein *Mp*IBP.

### 3.7. The adhesion of *Marinomonas primoryensis* to ice is prevented by blocking region IV of *Mp*IBP

To check if *M**.** primoryensis* binds ice through *Mp*IBP_RIV, we used antibodies raised against *Mp*IBP_RIV and against the 104-amino-acid repeats that make up *Mp*IBP_RII (anti-4xRII). The electronic supplementary material, movie S6, and figures 4 and 5 show that addition of anti*-Mp*IBP_RIV antibodies blocked the ability of *M. primoryensis* to bind ice. Addition of anti-4xR_II antibodies had no apparent effect on the ice binding (figure 5*c*). This emphasizes that *Mp*IBP_RIV is the domain responsible for adhesion to ice. To further verify this point, we tested the effect of the anti-sera on the TH and ice-shaping activity of recombinant *Mp*IBP_RIV. At 5 µM and 33% serum, we did not see a difference between the TH of *Mp*IBP_RIV in the presence of non-immunized serum, anti-*Mp*IBP_RIV antibody or anti-*Mp*IBP_RII antibody. However, at one-fifth of this *Mp*IBP_RIV concentration (1 µM) and 97% serum the sample containing anti*-Mp*IBP_RIV serum had no TH activity and almost no ice shaping, whereas the samples containing anti-4xRII or the non-immunized serum had a TH of approximately 1°C. This indicates that the anti-*Mp*IBP_RIV antibodies disrupt the ice-binding site (IBS) of *Mp*IBP_RIV and do not interfere indirectly with the ability of the bacteria to bind ice. The antibodies by themselves have no apparent TH or ice-shaping activity when tested without *Mp*IBP_RIV. Addition of the metal chelators EDTA or EGTA, the latter of which is more specific to Ca^2+^ ions, resulted in severe damage to the motility of the bacteria and it was difficult to compare the ice-binding properties of non-swimming bacteria with those of swimming ones.

**Figure 9.**
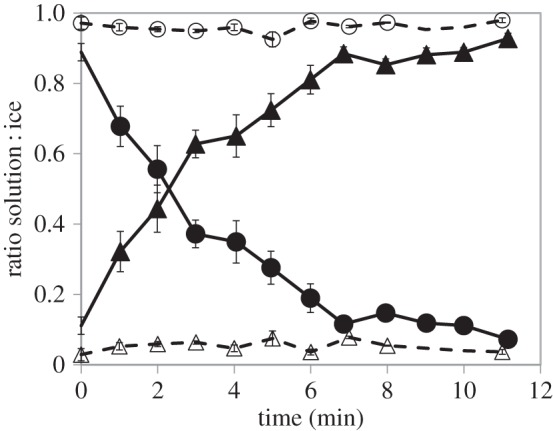
Analysis of *Marinomonas primoryensis* adhesion to ice over time and the effect of anti-*Mp*IBP_RIV. The plot presents the distribution of bacteria between ice and medium over time. The ratios of ice-bound versus free bacteria were measured over time in the presence of anti-*Mp*IBP_RIV serum (open circles and open triangles for solution and ice, respectively, dashed line) and pre-immune serum without anti-*Mp*IBP_RIV (filled circles and filled triangles for solution and ice, respectively, solid line). At an OD_600 nm_ of 0.02, it takes 3 min for half the population to bind ice. Experiments with non-immunized serum or without serum showed indistinguishable results. The data are based on 20 independent experiments. Up to 6 min, *n* = 8–10; minute 7 and above, *n* = 3–4. Error bars represent standard error.

We analysed the time it takes for a population of *M. primoryensis* to bind ice by counting the free bacteria in solution relative to surface-bound bacteria over time. Figure 9 shows the ratio of *M. primoryensis* bound to a single ice crystal and *M. primoryensis* free in solution. At time zero, there are few individuals on the ice surface. After only 3 min, half of the visible bacteria were already bound to the ice crystal. After 10 min approximately 90% of the visible bacteria were bound to the ice. Longer accumulation times were observed for lower bacteria concentrations and shorter times for dense populations (approx. 2 min for twofold concentration and 6 min for one-fifth). Addition of anti-*Mp*IBP_RIV serum to the solution reduced the attachment to ice to practically zero. The analysis was conducted on diluted bacteria solutions to be able to count the bound bacteria over 10 min of binding before the ice gets either too large or too crowded whereupon it becomes difficult to discriminate a single bacterium.

## 4. Discussion

*Marinomonas primoryensis* were first isolated from coastal sea ice in the sea of Japan [39] and then from the upper layer of the Ace and Pendant Lakes in Antarctica [24]. Using an IR inhibition assay, the presence of an IBP in these Antarctic isolates was shown. At that time, it was suggested that this activity might protect the bacterium from freezing damage. Further analysis of *Mp*IBP showed that this protein is not secreted into the medium [17], unlike many microorganism IBPs [40]. These findings emphasized that *Mp*IBP might have a different role in Nature from the DUF3494 bacterial IBPs, which seem to control the structure of ice surrounding the microorganism [40]. Other ice-active proteins were reported in bacteria from harsh environments where the temperature drops way below zero during winter such as Antarctic and cold temperate soils [35,41] or high Arctic plant rhizosphere [9]. At these temperatures, it is likely that freezing will be tolerated and that IBPs could serve to inhibit IR.

**Figure 10.**
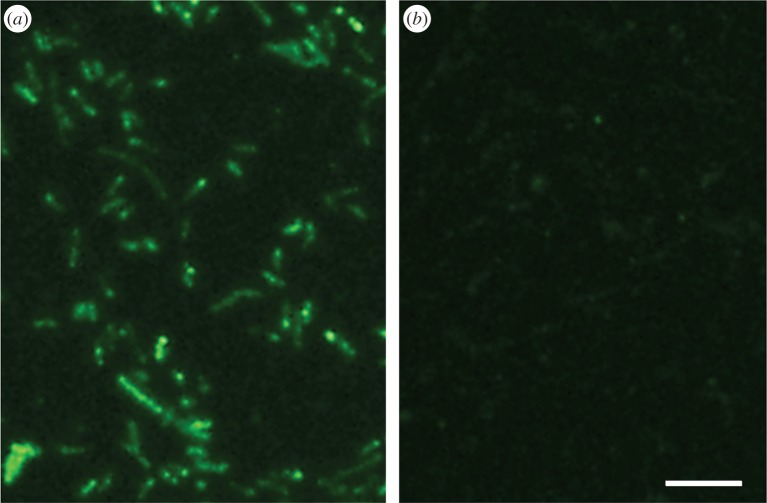
Immunofluorescence of *Mp*IBP. Immobilized *Marinomonas primoryensis* were immunolabelled with anti-*Mp*IBP_RIV serum and visualized using fluorescently labelled goat anti-rabbit second antibody. (*a*) Anti-*Mp*IBP_RIV serum. (*b*) Pre-immunized serum. Scale bar, 10 µm. Reaction with anti-MpIBP_RII anti-sera was previously demonstrated [19]. (Online version in colour.)

The Ace and Pendant Lakes are permanently cold with temperatures ranging between −1°C and +1°C and are covered by an ice layer of 1–2 m thickness throughout most of the year [24]. In this environment, the lake bacteria do not need to cope with extremely low temperatures. Evidence that the *Mp*IBP is located on the outer surface of the bacteria (figure 10) along with biochemical and bioinformatic studies [19] suggested that this protein is an adhesin that attaches the bacteria to ice surfaces. Here, we present direct evidence that *Mp*IBP is an ice adhesin. The ability of anti-*Mp*IBP_RIV antibodies to block ice-binding activity and the failure of anti-*Mp*IBPR_II antibodies to do so imply that the ice binding by the bacteria is indeed due to region IV of *Mp*IBP. It is thought that *Mp*IBP_RII serves as extender domains to help the bacteria reach out to the ice [23]. The binding of antibodies to this region is unlikely to prevent contact with ice.

Not all individual bacteria that contacted ice attached to it. We wonder if the bacteria may have some control over the exposure of their IBS that allows them to bind ice following a chemotaxis stimulus. Another possibility is that the orientation and competence of the IBS of the protein relative to the ice surface at the moment of contact might not allow binding. According to the anchored clathrate water mechanism [20], a molecule of *Mp*IBP will attach to an ice crystal when its IBS is bound to a quorum of ice-like waters and is positioned parallel to the ice surface. This condition can be fulfilled in solution when the protein is freely moving around by diffusion. With the protein located on the surface of a swimming bacterium, its orientation and time in contact with ice will be dictated by bacterial movement.

The ice-binding domain of *Mp*IBP has the ability to bind many ice planes, which might increase the probability of binding the bacteria to ice. However, *Mp*IBP_RIV has a strong preference for binding the basal planes in accordance with previous data including fluorescence ice plane affinity experiments [21], ice shaping [18,27], ice burst pattern [7] and the fact that this protein is hyperactive. Indeed, when the bacteria approached the basal plane, there seemed to be fewer incidents of ‘bouncing off’ the crystal, and these two surfaces accumulate the bacteria more efficiently than others. This might also reflect the stable nature of the basal plane with water adding to it more slowly than other planes of ice.

Directional movement of bacteria has long been attributed to chemotaxis [42] and thermotaxis [43,44], and has been seen in marine bacteria that need to cope with scarce nutrients and turbulence [45,46]. *Marinomonas primoryensis* are Gram-negative, strictly aerobic bacteria. It is not surprising that they are abundant at the upper layers of the water, where oxygen and nutrients are available. It is possible that the bacteria ‘sense’ the ice by some sort of thermotaxis and/or chemotaxis, i.e. change their swimming pattern due to a temperature or a chemical gradient associated with ice or sense signals from bacteria that are already attached to the ice. In our experiments, we observed a dramatic change in the swimming pattern with temperature, from a strategy of long ‘runs’ of over 100 µm and smooth turns at 0°C, to ‘run and reverse’ [42] with short runs of 10–20 µm and frequent abrupt reversals at 20°C. The observed change of swimming pattern with temperature can be a thermotaxis strategy or an artefact, as warm temperatures may not represent conditions that the bacteria face in their natural habitat. Growth at 20°C for 15 h resulted in bacteria that swam poorly and had defective cell divisions. Nevertheless, a ‘back and forth’ swimming strategy was shown to be beneficial in other marine bacteria for seeking nutrients and clustering around them in the open sea where food is scarce and flows and turbulence exist [46–49]. The fast reversals of movement help them to cluster around nutrients, and their rapid movement grants higher sensitivity to chemical gradients. The swimming strategy of *M. primoryensis* at 0°C is probably favourable for binding to ice, where the ice layer is thick, wide and stable, it is not significantly affected by flows, and the speed of the bacteria is slower. In Antarctica, *M*. *primoryensi*s live at close to 0°C all year long, but they have also been isolated from the upper layer of the Sea of Japan, which heats up to temperatures of 20°C or above in the summer [50]. *Marinomonas primoryensis* were collected from sea ice during winter in the Sea of Japan [39] and we do not know where they are found in summer.

Many studies have shown that the binding of IBPs to ice is irreversible [1,26,51,52]. However, we observed that bound bacteria are not incorporated into the ice during growth, and they do not prevent ice growth. Rather, they are pushed away by growing ice fronts that approach from the sides and overgrow the established ice front. In order to stop the growth, the accumulation rate of the bacteria (figure 9) should be much higher. In addition, the effective concentration of the *Mp*IBP is dictated by the concentration and orientation of the bacteria. Therefore, the idea of *Mp*IBP acting as an AFP is practically impossible. In ice-affinity purification, bound IBPs are overgrown and incorporated into the new ice because they are small single proteins. Larger structures, even multi-subunit complexes, tend to be sheared off by the growing ice. Thus, it is not surprising that the bacteria are not included into the ice. This is to the benefit of the bacteria because their incorporation into ice would promote their freezing damage or at least exclude the possibility of maintaining liquid around them, which is necessary for nutrient uptake. It is also quite possible that some *Mp*IBP are sheared off the surface of the bacterium and become incorporated into the ice.

The adhesion of *M. primoryensis* to ice may be the first step in biofilm formation. The observed microcolonies at the interface of the ice and the microfluidic channel can also be related to initial stages of biofilm growth. Biofilms are dynamic assemblages that take days to develop at room temperature [53,54], so the time frame for their development may be considerably longer at 0°C. Thus, it is not surprising that we did not observe clear interactions between individual bacteria.

The IBP (region IV) and extender domains (region II) of *Mp*IBP are dependent on calcium ions for their structural integrity. Therefore, we attempted to knock out ice binding by chelating calcium ions through the addition of EGTA to the solution. Loss of calcium drastically reduced the swimming ability of the bacteria. Bacteria that cannot swim did not reach the ice for binding, so the effect of EGTA on the binding activity could not be evaluated. Our attempts to abolish the ability of *M. primoryensis* to bind ice without affecting their swimming ability were successful using the anti-*Mp*IBP_RIV antibody. This is a significant result because it suggests that reagents that can interfere with the initial adsorption of bacteria to their substrate might completely block colonization and subsequent biofilm formation. This strategy could be applied to combat many systemic bacterial infections.

## 5. Conclusion

Our findings provide direct evidence that *Mp*IBP is an ice adhesin that serves to attach *M. primoryensis* to ice. When bound to its host bacterium, *Mp*IBP does not significantly affect the freezing or melting points of the ice. This remarkable adaptation of the adhesin principle used by bacteria to bind to many kinds of surfaces is currently the only example where an adhesin has evolved to bind ice. We show that a polyclonal antibody directed against the ice-binding domain of *Mp*IBP can completely prevent bacterial adhesion to its substrate. Such bacterial adhesion inhibitors are potentially useful for blocking bacterial attachment to other surfaces where biofilm formation is harmful.

## Supplementary Material

Supporting information
