# Supplementary material for: Putting life on ice: bacteria that bind to frozen water
Source: J R Soc Interface. 2016 Aug;13(121):20160210. doi: 10.1098/rsif.2016.0210 (PMC5014055; doi:10.1098/rsif.2016.0210)
Supplement: Supporting information [file rsif20160210supp1.pdf]

# Putting life on ice: Bacteria that bind to frozen water

Maya Bar Dolev[1], Reut Bernheim[1], Shuaiqi Guo[2], Peter L. Davies[2] and Ido Braslavsky[1*]

# Supporting information

**Control Bacteria used for comparison to *M. primoryensis***

The bacteria used for control in the study of the IBP from *M. primoryensis* include: *Planococcus halocryophilus*, a psychrophilic halophilic motile bacterium isolated from arctic permafrost [1] , **obtained from Lyle White (McGill University, Quebec, Canada)**; *Flavobacterium frigoris* [2,3] and the Vostok ice core bacterium, two psychrophilic bacteria that secrete IBPs , **obtained from James Raymond (University of Nevada, Las Vegas, NV)**. The Vostoc ice core bacterium was isolated from deep within the Vostok ice core from Antarctica [5], and *Flavobacterium frigoris* was isolated from microbial mats [4] and sea ice [2] in Antarctica. *Oleisphira antarctica*, an oil-degrading, motile marine bacterium isolated from Antarctic sea water [6] was obtained from **Peter N. Golyshin (Bangor University, Bangor, UK)**; *Vibrio shiloi, a* motile marine bacterium [7] received **from Eugene Rosenberg (Tel Aviv University, Tel Aviv, Israel);** and *E. coli* (strain CP875), obtained from Michael Eisenbach (Weizmann Institute of Science, Rehovot, Israel).

**Growth conditions**

*E. coli* (CP875) was grown either at 4 °C for 2 days without shaking or over-night at 20 °C with shaking of ~150 rpm to log phase, in both marine broth and Luria broth.

*Vibrio shiloi* was grown in marine broth over night at 20 - 25 °C or in LB at 130 rpm to mid-log phase.

The bacteria isolated from Vostock core were grown in LB, marine broth or 3% tryptic soy broth (TSB) at 0 – 4 °C without shaking

*Planococus halocryophilus* were grown in 10, 15 or 20% TSB supplemented with 0, 5 or 10% NaCl . The bacteria were inspected after 2,3 and 5 days of growth at 4 °C or 10 °C without shaking. We also tried to grow them in 3% TSB.

*Flavobacterium frigoris* were grown in 50% marine broth at 0 – 4 °C without shaking and assayed adter 3, 5 and 7 days.

*Pseudomonas* species (*p. fluorescens, p. borealis* and *p. syringae*) were grown on 3% and 10% TSB for 3 days at 4 °C and over-night at 16 °C without shaking , to mid-log phase. We also tried to replace the medium with LB.

*Oleisphira antarctica* were grown on ONR7a medium (self prepared according to https://www.dsmz.de/microorganisms/medium/pdf/DSMZ_Medium950.pdf) supplemented with 0.5% Tween-40 or n-tetradecane for 5-20 days at 4-16 °C. We also tried to enrich the medium with trace element solution SL-10 (https://www.dsmz.de/microorganisms/medium/pdf/DSMZ_Medium461.pdf).

**References**


1. Mykytczuk NC, Wilhelm RC, Whyte LG (2012) Planococcus halocryophilus sp. nov., an extreme sub-zero species from high Arctic permafrost. Int J Syst Evol Microbiol 62: 1937-1944.
2. Do H, Lee JH, Lee SG, Kim HJ (2012) Crystallization and preliminary X-ray crystallographic analysis of an ice-binding protein (FfIBP) from Flavobacterium frigoris PS1. Acta Crystallographica Section F: Structural Biology and Crystallization Communications 68: 806-809.
3. Do H, Kim SJ, Kim HJ, Lee JH (2014) Structure-based characterization and antifreeze properties of a hyperactive ice-binding protein from the Antarctic bacterium Flavobacterium frigoris PS1. Acta Crystallogr D Biol Crystallogr 70: 1061-1073.
4. Van Trappen S, Vandecandelaere I, Mergaert J, Swings J (2004) Flavobacterium degerlachei sp. nov., Flavobacterium frigoris sp. nov. and Flavobacterium micromati sp. nov., novel psychrophilic bacteria isolated from microbial mats in Antarctic lakes. International Journal of Systematic and Evolutionary Microbiology 54: 85-92.
5. Achberger AM, Brox TI, Skidmore ML, Christner BC (2011) Expression and Partial Characterization of an Ice-Binding Protein from a Bacterium Isolated at a Depth of 3,519 m in the Vostok Ice Core, Antarctica. Frontiers in Microbiology 2: 255.
6. Yakimov MM, Giuliano L, Gentile G, Crisafi E, Chernikova TN, et al. (2003) Oleispira antarctica gen. nov., sp. nov., a novel hydrocarbonoclastic marine bacterium isolated from Antarctic coastal sea water. Int J Syst Evol Microbiol 53: 779-785.
7. Kushmaro A ,Banin E, Loya Y, Stackebrandt E, Rosenberg E (2001) Vibrio shiloi sp nov., the causative agent of bleaching of the coral Oculina patagonica. International Journal of Systematic and Evolutionary Microbiology 51: 1383-1388.

## Figure S1

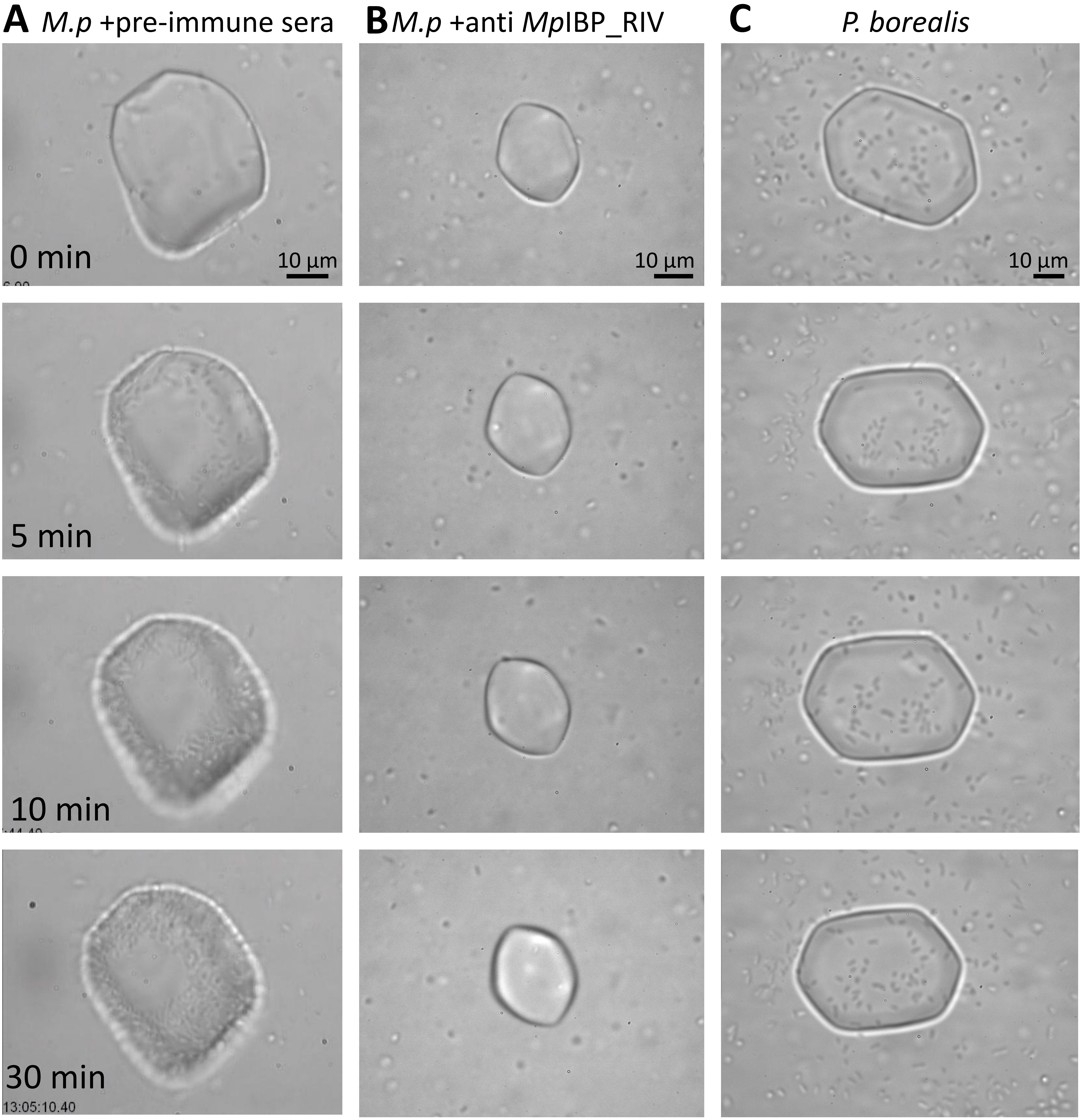


**Accumulation of *M. primoryensis* o ice in the presence of AFPs**. (A) 0.1 µM of *Tm*AFP was added to the solution. The AFP stabilizes the crystal and the bacteria are concentrated on its surface over time. (B) Addition of anti *Mp*IBP_RIV antibodies precludes the binding. (C) *P. borealis* do not adhere to ice. The bacteria visible on the ice are swimming on top of it or non-motile individuals that float.

## Figure S2

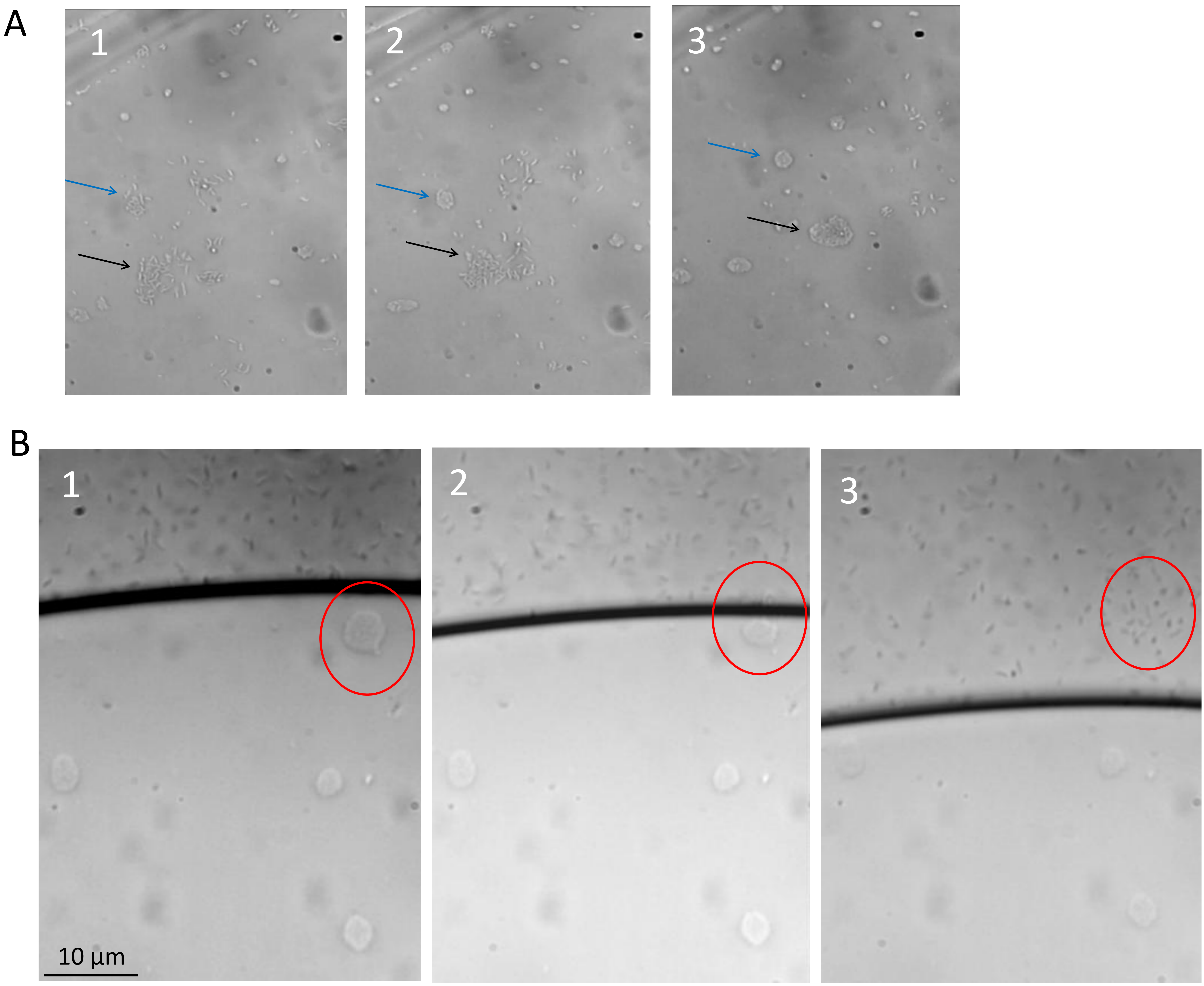


**Bacteria form patches on the ice**. Bacteria entrapped at the interface between the ice and the PDMS form condensed patches. (A) A sequence showing the patch formation from left to right. Two arrows show two patches. (B) A sequence showing patch movement during ice melting. When the ice grows or melt the patch advances to the ice front as a unit. When it reches the ice front the bacteria are released to solution and swim as before the ice growth. A melting patch is marked by a red oval and followed. Sea movies S4 and S10 for a better view.
